# 重组人红细胞生成素联合环孢素A治疗获得性纯红细胞再生障碍的疗效与安全性

**DOI:** 10.3760/cma.j.issn.0253-2727.2021.11.011

**Published:** 2021-11

**Authors:** 泽松 陈, 辰 杨, 苗 陈, 冰 韩

**Affiliations:** 中国医学科学院北京协和医学院、北京协和医院血液科 100730 Department of Hematology, Peking Union Medical College Hospital, Chinese Academy of Medical Sciences and Peking Union Medical College, Beijing 100730, China

纯红细胞再生障碍（PRCA）是以骨髓红系造血衰竭为特征的疾病[1]。原发性PRCA治疗包括免疫抑制剂和支持治疗[2]。多种免疫抑制剂均可治疗PRCA，环孢素A（CsA）是一线选择，单药有效率为65％～87％[3]–[4]。对症支持治疗包括输血、促造血、去铁治疗等。已有个案报道显示免疫抑制剂联合促造血药物，如雄激素、重组人红细胞生成素（rhEPO），能够使患者更早脱离输血依赖，更快达到疾病缓解[5]–[7]。免疫抑制治疗起效前PRCA患者常有严重输血依赖，如能在治疗早期快速改善贫血可显著提高患者生活质量。本研究我们回顾性分析59例原发性PRCA患者的临床资料，比较了CsA单药和CsA+rhEPO治疗PRCA的疗效和安全性。

## 病例与方法

1. 病例：回顾性分析2015年1月至2020年1月我院确诊的142例获得性PRCA患者。纳入标准：①符合原发获得性PRCA诊断标准[1]；②18岁以上；③HGB<100 g/L；④接受免疫抑制剂治疗≥6个月且随访≥1年，疗效可评价；⑤签署知情同意书。排除标准：①治疗前C反应蛋白>20 mg/L或合并感染、肾功能异常、中重度系统性疾病；②接受过其他药物治疗，如糖皮质激素或其他免疫抑制剂；③数据不完整或患者不同意提供临床数据。最终纳入接受CsA±rhEPO治疗的原发获得性PRCA患者59例，其中CsA+rhEPO治疗（CsA+rhEPO组）患者44例，基线情况匹配、仅接受CsA单药治疗（CsA单药组）患者15例。

2. 治疗方案：CsA：3～5 mg·kg^−1^·d^−1^，监测血药浓度维持谷浓度100～200 µg/L。有效（OR）者至少治疗18个月，6个月内减量至停药。rhEPO：与CsA同时开始，10 000 U隔日1次皮下注射，直至疗效达完全缓解（CR），未达CR者rhEPO应用至少3个月。HGB<60 g/L时给予红细胞输注。随访期内所有患者均未接受去铁治疗。

3. 定义与疗效评估标准：HGB≤60 g/L定义为输血依赖。CR：HGB≥120 g/L（男性）或110 g/L（女性），骨髓造血恢复正常；部分缓解（PR）：HGB较基线升高≥30 g/L，脱离输血，未达到CR；OR：CR+PR；无效（NR）：输血依赖或HGB较基线升高<30 g/L。起效时间为开始治疗至达到OR的时间间隔。

4. 数据采集及随访：收集患者治疗前血常规、血生化、铁蛋白、免疫指标、病毒指标、骨髓活检、染色体核型、髓系肿瘤相关基因检测及必要的影像学检查等，除外其他贫血性疾病及可能的继发因素。记录治疗1、2、3、6、12个月及随访终点的症状、实验室检查及不良反应情况，详细记录患者疾病转化、转归等指标。随访截止时间为2020年12月。CsA+rhEPO组患者中位随访19（12～30）个月，CsA单药组患者中位随访23（16～30）个月。

5. 统计学处理：使用SPSS 25.0进行数据分析。连续变量采用独立样本*t*检验（服从正态分布）或Mann-Whitney *U*检验（不服从正态分布）进行两组间比较；分类变量采用Fisher精确概率法进行差异性比较。*P*<0.05为差异有统计学意义。

## 结果

1. 基线临床特征：见表1，59例原发获得性PRCA患者中男31例，女28例，中位年龄58（49～70）岁。两组患者治疗前各临床特征差异均无统计学意义。

**表1:** 获得性纯红细胞再生障碍患者基线临床特征比较

临床特征	总体（59例）	CsA+rhEPO组（44例）	CsA单药组（15例）	*P*值
中位年龄［岁，*M*（范围）］	58（49～70）	64（53～69）	56（47～75）	0.767
性别（例，女/男）	28/31	18/26	10/5	0.087
HGB（g/L，*x±s*）	65.2±4.0	65.2±4.9	65.1±7.4	0.980
输血依赖［例（％）］^a^	31（52.5）	22（50.0）	9（60.0）	0.561
Ret（×10^9^/L，*x±s*）	12.7±1.7	13.0±2.1	11.7±2.7	0.503
ALT（U/L，*x±s*）	22.3±3.0	23.2±3.7	19.5±5.3	0.296
血肌酐（µmol/L，*x±s*）	77.2±6.0	77.1±7.1	77.5±12.7	0.955
铁蛋白［µg/L，*M*（范围）］	672（383～931）	638（377～902）	774（400～1 161）	0.330
CsA治疗时间［月，*M*（范围）］	21（15～24）	22（15～24）	19（12～24）	0.504
rhEPO治疗时间［月，*M*（范围）］	NA	2.5（2～6）	NA	NA

注：CsA：环孢素A；rhEPO：重组人红细胞生成素；NA：不适用。a：HGB≤60 g/L定义为输血依赖

2. 疗效分析：两组患者总体疗效比较见表2。CsA+rhEPO组达到最佳疗效的中位时间显著短于CsA单药组［2（1～4）个月对3（3～6）个月，*z*＝−2.061，*P*＝0.045］，CsA+rhEPO组起效时间短于CsA单药组，但差异无统计学意义［1（1～3）个月对3（2～3）个月，*z*＝−1.946，*P*＝0.066］。CsA+rhEPO组输血依赖患者（22例）中位输血时间为1（1～3）个月CsA单药组（9例）中位输血时间为2（1～3）个月，差异无统计学意义（*z*＝−1.043，*P*＝0.379）。

**表2:** 不同治疗组纯红细胞再生障碍患者各时间下疗效比较［例数（％）］

组别	例数	治疗1个月			治疗2个月			治疗3个月		
		CR	PR	OR	CR	PR	OR	CR	PR	OR
CsA+rhEPO组	44	12（27.3）	6（13.6）	18（40.9）	18（40.9）	5（11.4）	23（52.3）	23（52.3）	7（15.9）	30（68.2）
CsA单药组	15	0（0.0）	2（13.3）	2（13.3）	0（0.0）	3（20.0）	3（20.0）	4（26.7）	5（33.3）	9（60.0）

*P*值		0.026	1.000	0.064	0.002	0.407	0.038	0.133	0.263	0.763

组别	例数	治疗6个月			治疗12个月			随访终点		
		CR	PR	OR	CR	PR	OR	CR	PR	OR
CsA+rhEPO组	44	26（59.1）	6（13.6）	32（72.7）	25（56.8）	9（20.5）	34（77.3）	21（47.7）	8（18.2）	29（65.9）
CsA单药组	15	7（46.7）	2（13.3）	9（60.0）	8（53.3）	1（6.7）	9（60.0）	7（46.7）	1（6.7）	8（53.3）

*P*值		0.549	1.000	0.517	1.000	0.426	0.312	1.000	0.424	0.537

注：CsA：环孢素A；rhEPO：重组人红细胞生成素；CR：完全缓解；PR：部分缓解；OR：有效

治疗后1个月，CsA+rhEPO组CR率显著高于CsA单药组（27.3％对0，*P*＝0.026），两组PR率、OR率差异无统计学意义。治疗后2个月，CsA+rhEPO组CR率（40.9％对0，*P*＝0.002）、OR率（52.3％对20.0％，*P*＝0.038）均显著高于CsA单药组，PR率差异无统计学意义。治疗后3、6、12个月及随访终点，CsA+rhEPO组与CsA单药组在CR、PR、OR率上差异均无统计学意义。

3. 两组HGB水平比较：不同治疗时间下两组患者平均HGB水平恢复情况见表3。治疗前两组平均HGB水平差异无统计学意义（*P*＝0.980），治疗1个月后rhEPO组显著高于对照组［（94.7±8.2）g/L对（80.4±10.0）g/L，*P*＝0.026］。治疗2、3、6、12个月及随访终点两组平均HGB水平差异均无统计学意义（*P*值均>0.05）。

**表3:** 不同治疗组纯红细胞再生障碍患者血红蛋白水平比较（g/L，*x±s*）

组别	例数	治疗前	治疗1个月	治疗2个月	治疗3个月	治疗6个月	治疗12个月	随访终点
CsA+rhEPO组	44	65.2±4.9	94.7±8.2	101.8±8.8	110.5±8.6	112.6±8.6	117.9±8.4	119.8±9.2
CsA单药组	15	65.1±7.4	80.4±10.0	90.3±9.7	101.1±18.4	105.2±15.7	113.6±16.0	114.9±20.3

*P*值		0.980	0.026	0.074	0.340	0.400	0.617	0.630

注：CsA：环孢素A；rhEPO：重组人红细胞生成素

4. 不良反应：治疗期间出现的药物相关不良事件见表4，包括感染、肝损伤、肾损伤、齿龈增生及注射部位疼痛。CsA+rhEPO组涉及以上所有种类，对照组仅出现感染、肝损伤、肾损伤。5例感染病例中4例为肺部感染，经CsA减量联合抗感染治疗好转；1例为肛周脓肿，后放弃CsA治疗。其他不良反应经CsA减量或对症治疗后均有所好转。在随访期内，两组均未进展为骨髓增生异常综合征（MDS）/急性髓系白血病（AML）。

**表4:** 不同治疗组纯红细胞再生障碍患者不良反应比较［例数（％）］

组别	例数	感染	肝损伤	肾损伤	齿龈增生
CsA+rhEPO	44	3（6.8）	3（6.8）	4（9.1）	2（4.5）
CsA单药	15	2（13.3）	2（13.3）	1（6.7）	0（0.0）

*P*值		0.593	0.593	1.000	1.000

注：CsA：环孢素A；rhEPO：重组人红细胞生成素

5. 随访结局：随访终点全部患者总体的CR率为47.5％，OR率为62.7％，复发率为22.2％（10/45）。在随访期内，无一例病例死亡。CsA+rhEPO组共有6例（17.6％）在治疗有效后复发：4例为CsA减量复发，经CsA加量或换用其他治疗后均再次缓解；1例为CsA减量复发，多种治疗后仍未缓解；1例CsA足量治疗时复发，重新评估后确诊为B细胞淋巴瘤，现原发病治疗中。CsA单药组共4例（36.3％）在治疗有效后复发：2例为CsA减量复发、1例为CsA停药复发，经CsA加量或换用其他治疗后均再次缓解；1例治疗1年出现肛周脓肿并放弃CsA治疗。两组患者复发率差异无统计学意义（*P*＝0.228）。

## 讨论

EPO能够调控红系造血细胞存活、增殖、分化、成熟，促进骨髓造血[8]。其通过与骨髓红系祖细胞表面EPO受体结合，激活相关信号通路发挥作用[9]。rhEPO与内源性EPO生物活性相当，已被广泛应用于多种贫血及骨髓衰竭性疾病的治疗中[8],[10]。

PRCA是以骨髓红系造血衰竭为主要特征的疾病，目前多认为其发病机制与免疫异常相关[11]。以CsA为主的免疫抑制治疗是临床常用疗法，缓解率较高[1]–[2],[12]。然而CsA起效时间长，通常需要数月不等[13]，起效前患者常受重度贫血困扰，表现为严重输血依赖。缩短起效时间，使PRCA患者更早脱离输血依赖，可能改善其生活质量。

已有个案报道称，CsA联合rhEPO能够缩短起效时间、减少输血量、提高生存质量[5]–[6],[14]。但由于疾病罕见、治疗方案多样，难以进行基线匹配且治疗较为统一的对照研究，目前国内外尚无探索EPO疗效的大样本研究。为进一步探究CsA联合rhEPO治疗方案的可行性，我们回顾性分析了CsA+rhEPO治疗获得性PRCA患者的临床数据，并与基线相近的同期使用CsA单药治疗患者的临床数据进行对比。虽然例数有限，但依然为目前样本量最大、随访时间较长且数据较为完整的对照研究。

相较于CsA单药组，CsA+rhEPO组患者达到最佳疗效所需时间显著缩短。在治疗早期，CsA+rhEPO组患者的CR率、OR率和平均HGB水平优于对照组。随治疗时间延长，CsA效果逐步显现，同时多数患者停用rhEPO，两组在CR、PR、OR率及平均HGB水平上的差异逐渐消失。提示CsA联合rhEPO治疗能够在短时间内提高患者HGB水平，加速疾病缓解，使患者尽快脱离输血，从而改善生活质量。在治疗起效后，停止rhEPO治疗并不会影响远期疗效。此外，疾病快速缓解有助于减少输血量，降低输血风险，提高患者治疗疾病的信心和依从性。

不良反应方面，除注射部位疼痛外，其他不良反应均与CsA相关性较大，联用rhEPO并未明显增加不良反应发生率。需要注意的是，长期应用rhEPO可介导机体产生抗EPO抗体，继发PRCA[15]–[16]。目前认为该效应与佐剂和注射器相关[15],[17]，改进后的剂型已很少导致PRCA的发生[18]。本研究中大部分患者rhEPO治疗时间较短，未见相关不良反应。原发性PRCA是良性疾病，发生MDS/AML转化很少，本研究也未发现联用rhEPO增加上述克隆演变的作用。

由于PRCA发病率低，相当数量的患者为继发性，治疗方案也存在差异，本研究总体例数偏少。此外，很多患者并未在治疗开始前检测血清EPO水平，因此在可能影响rhEPO疗效因素方面，我们无法得出可靠结论。尽管如此，本研究进一步证实，CsA联合rhEPO治疗可能给PRCA患者带来更多益处。未来需要设计良好的、更大样本量的前瞻对照研究将得出更可靠的结论。

本回顾性研究发现，与CsA单药相比，CsA联合rhEPO方案在治疗第1、2个月具有更高的缓解率，平均HGB水平上升更为明显，且未明显增加不良事件发生率。因此，PRCA患者在开始CsA治疗的同时启动rhEPO治疗，可能使患者更早脱离贫血而获益。
